# Supplementary material for: Gender-related disparities in the frequencies of PD-1 and PD-L1 positive peripheral blood T and B lymphocytes in patients with alcohol-related liver disease: a single center pilot study
Source: PeerJ. 2021 Jan 20;9:e10518. doi: 10.7717/peerj.10518 (PMC7825365; doi:10.7717/peerj.10518)

# **Supplementary Information file**

**Title of the manuscript: Gender-related disparities in the frequencies of PD-1 and PD-L1 positive peripheral blood T and B lymphocytes in patients with alcohol-related liver disease- a single center pilot study.**

### Beata Kasztelan-Szczerbinska^1*^, Katarzyna Adamczyk^1^, Agata Surdacka^2^, Halina Cichoz-Lach^1^, Jacek Rolinski^2^, Agata Michalak^1^, Agnieszka Bojarska- Junak^2^, Mariusz Szczerbinski^3^

### ^1^ Department of Gastroenterology with the Endoscopy Unit, Medical University of Lublin, Poland,

### ^2^ Department of Clinical Immunology, Medical University of Lublin, Poland,

### ^3^ Public, Academic Hospital number 4, Department of Gastroenterology with the Endoscopy Unit, Lublin, Poland

### * the corresponding author

**Supplementary Table** **S1.** Comparison of the frequencies (%) of PD-1/PD-L1 positive T and B lymphocytes in ALD patients versus controls (^a^Mann-Whitney test).

| Variable | **ALD group (n=56)** | | **Controls (n=25)** | |  |
| --- | --- | --- | --- | --- | --- |
|  | Median | 5–95 Percentile | Median | 5–95 Percentile | p ^a^ |
| **PD-1 positive cell subsets** | | | | | |
| **CD4+** | 18.44 | 6.03 - 50.43 | 19.72 | 7.69 - 36.08 | 0.54 |
| **CD8+** | 21.17 | 7.02 - 46.30 | 24.28 | 8.31 - 40.48 | 0.18 |
| **CD19+** | 3.76 | 0.85 - 24.03 | 2.51 | 0.61 - 17.12 | 0.10 |
| **PD-L1 positive cell subsets** | | | | | |
| **CD4+** | 0.74 | 0.08 - 6.75 | 1.18 | 0.13 - 4.93 | 0.29 |
| **CD8+** | 0.52 | 0.03 - 3.58 | 0.90 | 0.15 - 2.63 | 0.23 |
| **CD19+** | 0.97 | 0.01 - 10.16 | 0.53 | 0.09 - 6.52 | 0.26 |

**Supplementary** **Table** **S2.** Comparison of the frequencies (%) of PD-1/PD-L1 positive T and B lymphocytes in ALD patients with different CTP classes versus controls. ^a b^

| Variable | **ALD group** | | | | | | | **P1^a^** | **Controls**  **(n=25)** | | **P2^b^** | **P3^b^** | **P4^b^** |
| --- | --- | --- | --- | --- | --- | --- | --- | --- | --- | --- | --- | --- | --- |
|  | **CTP class** **A (n=14)** | | | **CTP class B (n=25)** | | **CTP class C (n=17)** | |  |  |  |  |  |  |
|  | Median | | 5 - 95  Percentile | Median | 5 - 95  Percentile | Median | 5 - 95  Percentile |  | Median | 5 - 95  Percentile |  |  |  |
| **PD-1 positive cell subsets** | | | | | | | | | | | | | |
| **CD4+** | 21.30 | | 10.59 - 86.72 | 14.42 | 0.70 - 30.13 | 19.71 | 5.03 - 35.39 | 0.10 | 19.72 | 7.69 - 36.08 | 0.54 | 0.08 | 1,00 |
| **CD8+** | 22.72 | | 5.53 - 70.86 | 17.34 | 0.73 - 63.33 | 23.88 | 4.70 - 71.52 | 0.27 | 24.28 | 8.31 - 40.48 | 0.70 | 0.03 | 0.73 |
| **CD19+** | 5.49 | | 0.69 - 18.86 | 3.15 | 0.57 - 11.53 | 4.57 | 0.48 - 22.38 | 0.20 | 2.51 | 0.61 - 17.12 | 0.09 | 0.51 | 0.03 |
| **PD-L1 positive cell subsets** | | | | | | | | | | | | | |
| **CD4+** |  | 0.59 | 0.02 - 16.31 | 0.74 | 0.16 - 7.62 | 0.83 | 0.03 - 10.51 | 0.74 | 1.18 | 0.13 - 4.93 | 0.40 | 0.28 | 0.78 |
| **CD8+** |  | 0.65 | 0.01 - 3.24 | 0.50 | 0.047 - 2.43 | 0.80 | 0.08 - 7.63 | 0.70 | 0.90 | 0.15 - 2.63 | 0.37 | 0.20 | 0.76 |
| **CD19+** |  | 0.60 | 0.01 - 23.84 | 0.92 | 0.01 - 11.82 | 1.30 | 0.01 - 18.37 | 0.83 | 0.53 | 0.09 - 6.52 | 0.59 | 0.38 | 0.18 |

^a^Kruskal-Wallis test with post-hoc analysis (Dunn); p1^a^ - CTP class A vs CTP class B vs CRP class C;

^b^Mann-Whitney test; p2^b^ - CTP class A vs controls; p3^b^ - CTP class B vs controls; p4^b^ - CTP class C vs controls

**Supplementary Table** **S3.** Comparison of the frequencies (%) of PD-1/PD-L1 positive T and B lymphocytes in ALD females with different CTP classes versus controls. ^a b^

| Variable | **ALD females** | | | | | | **P1^a^** | **Female controls**  **(n=9)** | | **P2^b^** | **P3^b^** | **P4^b^** |
| --- | --- | --- | --- | --- | --- | --- | --- | --- | --- | --- | --- | --- |
|  | **CTP class** **A (n=5)** | | **CTP class B (n=9)** | | **CTP class C (n=4)** | |  |  |  |  |  |  |
|  | Median | 5 - 95 Percentile | Median | 5 - 95 Percentile | Median | 5 - 95 Percentile |  | Median | 5 - 95 Percentile |  |  |  |
| **PD-1 positive cell subsets** | | | | | | | | | | | | |
| **CD4+** | 28.21 | 12.50 - 99.40 | 13.29 | 0.57 - 24.27 | 28.74 | 20.54 - 33.45 | 0.01 | 18.07 | 1.43 - 4.03 | 0.11 | 0.26 | 0.07 |
| **CD8+** | 25.35 | 21.09 - 30.64 | 10.13 | 0.59 - 31.38 | 33.24 | 16.19 - 65.89 | 0.07 | 23.06 | 0.67 - 6.70 | 0.52 | 0.08 | 0.50 |
| **CD19+** | 5.83 | 0.640 - 12.81 | 4.86 | 0.76 - 14.63 | 13.34 | 4.22 - 24.84 | 0.22 | 7.08 | 0.69 - 0.76 | 0.19 | 0.26 | 0.02 |
| **PD-L1 positive cell subsets** | | | | | | | | | | | | |
| **CD4+** | 2.86 | 0.08- 18.77 | 0.59 | 0.11 - 16.94 | 6.05 | 0.83 - 11.94 | 0.18 | 0.90 | 0.09 - 8.04 | 0.70 | 0.89 | 0.0503 |
| **CD8+** | 1.38 | 0.04 - 3.62 | 0.42 | 0.01 - 2.77 | 3.77 | 1.20 - 8.88 | 0.03 | 1.14 | 0.02 - 7.21 | 0.61 | 0.49 | 0.03 |
| **CD19+** | 3.71 | 0.01 - 25.09 | 0.92 | 0.01 - 31.40 | 8.63 | 1.47 - 21.01 | 0.30 | 1.99 | 0.01 - 8.88 | 0.18 | 0.13 | 0.006 |

^a^Kruskal-Wallis test with post-hoc analysis (Dunn); p1^a^ – ALD females : CTP class A vs CTP class B vs CTP class C;

^b^Mann-Whitney test; p2^b^ - ALD females with CTP class A vs controls; p3^b^ - ALD females with CTP class B vs controls; p4^b^ - ALD females with CTP class C vs controls

**Supplementary Table** **S4.** Comparison of the frequencies (%) of PD-1/PD-L1 positive T and B lymphocytes in ALD patients with CTP class A versus controls based on patients’gender.*

| **Variable** | **ALD and CTP class A** | | | | **p1*** | **Controls** | | | | **p2*** | **p3*** |
| --- | --- | --- | --- | --- | --- | --- | --- | --- | --- | --- | --- |
|  | **Males (n=9)** | | **Females (n=5)** | |  | **Males (n=16)** | | **Females (n=9)** | |  |  |
|  | Median | 5 - 95  percentile | Median | 5 - 95  percentile |  | Median | 5 - 95  percentile | Median | 5 - 95  percentile |  |  |
| **PD-1 positive cell subsets** | | | | | | | | | | | |
| **CD4+** | 18.00 | 10.11 - 29.60 | 28.21 | 12.50 - 99.40 | 0.11 | 21.07 | 8.40 - 9.64 | 14.20 | 6.55 - 30.67 | 0.36 | 0.11 |
| **CD8+** | 15.68 | 4.43 - 78.32 | 25.35 | 21.09 - 30.64 | 0.44 | 25.57 | 7.55 - 7.51 | 21.11 | 11.28 - 43.08 | 0.52 | 0.52 |
| **CD19+** | 5.35 | 0.91 - 20.37 | 5.83 | 0.64 - 12.81 | 0.36 | 2.54 | 0.78 - 9.39 | 1.66 | 0.35 - 14.70 | 0.30 | 0.19 |
| **PD-L1 positive cell subsets** | | | | | | | | | | | |
| **CD4+** | 0.38 | 0.01 - 6.47 | 2.86 | 0.08 - 18.77 | 0.24 | 1.06 | 0.26 - 5.10 | 1.83 | 0.10 - 4.03 | 0.14 | 0.70 |
| **CD8+** | 0.19 | 0.01 - 1.39 | 1.38 | 0.04 - 3.62 | 0.14 | 0.97 | 0.13 - 2.43 | 0.89 | 0.18 - 2.88 | 0.11 | 0.61 |
| **CD19+** | 0.57 | 0.01 - 2.06 | 3.71 | 0.01 - 25.09 | 0.29 | 0.69 | 0.25 - 8.77 | 0.45 | 0.07 - 1.52 | 0.51 | 0.18 |

* Mann-Whitney test; p1- ALD males with CTP class A vs ALD females with CTP class A; p2- ALD males with CTP class A vs male controls; p3- ALD females with CTP class A vs female controls

**Supplementary Table** **S5.** Comparison of the frequencies (%) of PD-1/PD-L1 positive T and B lymphocytes in ALD patients with CTP class B versus controls based on patients’gender.

| **Variable** | **ALD and CTP class B** | | | | **p1*** | **Controls** | | | | **p2*** | **p3*** |
| --- | --- | --- | --- | --- | --- | --- | --- | --- | --- | --- | --- |
|  | **Males (n=16)** | | **Females (n=9)** | |  | **Males (n=16)** | | **Females (n=9)** | |  |  |
|  | Median | 5 - 95  percentile | Median | 5 - 95  percentile |  | Median | 5 - 95  Percentile | Median | 5 - 95  Percentile |  |  |
| **PD-1 positive cell subsets** | | | | | | | | | | | |
| **CD4+** | 20.59 | 0.83 - 30.23 | 13.29 | 0.57 - 24.27 | 0.14 | 21.07 | 8.40 - 9.64 | 14.20 | 6.55 - 30.67 | 0.22 | 0.26 |
| **CD8+** | 18.29 | 1.76 - 77.97 | 10.13 | 0.59 - 31.38 | 0.19 | 25.57 | 7.55 - 7.51 | 21.11 | 11.28 - 43.08 | 0.16 | 0.08 |
| **CD19+** | 3.08 | 0.39- 7.84 | 4.86 | 0.76 - 14.63 | 0.28 | 2.54 | 0.78 - 9.39 | 1.66 | 0.35 - 14.70 | 0.84 | 0.26 |
| **PD-L1 positive cell subsets** | | | | | | | | | | | |
| **CD4+** | 0.96 | 0.21 - 2.57 | 0.59 | 0.11 - 16.94 | 0.91 | 1.06 | 0.26 - 5.10 | 1.83 | 0.10 - 4.03 | 0.34 | 0.89 |
| **CD8+** | 0.55 | 0.08 - 2.15 | 0.42 | 0.01 - 2.77 | 0.80 | 0.97 | 0.13 - 2.43 | 0.89 | 0.18 - 2.88 | 0.26 | 0.49 |
| **CD19+** | 1.09 | 0.06 - 4.59 | 0.92 | 0.01 - 31.40 | 0.86 | 0.69 | 0.25 - 8.77 | 0.45 | 0.07 - 1.52 | 0.89 | 0.13 |

* Mann-Whitney test; p1- ALD males with CTP class B vs ALD females with CTP class B; p2- ALD males with CTP class B vs male controls; p3- ALD females with CTP class B vs female controls

**Supplementary Table S6.** Comparison of the frequencies (%) of PD-1 and PDL-1 positive T and B cells in ALD patients with and without ascites versus controls (^a^ Mann-Whitney test).*

| **Variable** | **ALD patients with ascites**  **(n=26)** | | **ALD patients without ascites**  **(n=30)** | | p1^a^ | **Controls**  **(n=25)** | | p2^a^ | p3^a^ |
| --- | --- | --- | --- | --- | --- | --- | --- | --- | --- |
|  | Median | 5-95 percentiles | Median | 5-95 percentiles |  | Median | 5-95 percentiles |  |  |
| **PD-1 positive cell subsets** | | | | | | | | | |
| **CD4+** | 19.71 | 0.98 to 34.75 | 17.85 | 2.71 to 35.98 | 0.86 | 17.66 | 5.27 - 39.77 | 0.62 | 0.56 |
| **CD8+** | 21.34 | 3.49 to 67.50 | 21.17 | 0.78 to 78.32 | 0.80 | 19.72 | 7.69 - 36.08 | 0.20 | 0.29 |
| **CD19+** | 3.78 | 0.10 to 18.28 | 3.61 | 0.91 to 12.81 | 0.97 | 24.28 | 8.31 - 40.48 | 0.25 | 0.09 |
| **PD-L1 positive cell subsets** | | | | | | | | | |
| **CD4+** | 0.83 | 0.25 to 12.94 | 0.57 | 0.01 to 6.47 | 0.09 | 1.30 | 0.08 - 3.78 | 0.94 | 0.09 |
| **CD8+** | 0.78 | 0.12 to 5.54 | 0.42 | 0.01 to 2.32 | 0.08 | 1.18 | 0.13 - 4.93 | 0.78 | 0.08 |
| **CD19+** | 1.54 | 0.22 to 23.09 | 0.58 | 0.01 to 18.84 | 0.03 | 0.90 | 0.15 - 2.63 | 0.02 | 0.86 |

*p1- ALD patients with ascites vs ALD patients without ascites; p2- ALD patients with ascites vs controls; p3- ALD patients without ascites vs controls

**Supplementary Table S7:** Mutual correlations of the frequencies (%) of PD-1 and PD-L1 positive lymphocytes in the control group. (n=25; Rho- Spearman rank correlation coefficient).

|  | | **PD-1 positive cell subsets** | | | **PDL-1 positive cell subsets** | | |
| --- | --- | --- | --- | --- | --- | --- | --- |
|  | | **CD4+** | **CD8+** | **CD19+** | **CD4+** | **CD8+** | **CD19+** |
| **PD-1 positive** cell subsets | |  | | | | | |
| **CD4+** | Rho  P |  | 0.67 0.0002 | 0.30 0.15 | 0.04 0.86 | -0.36 0.08 | -0.03 0.90 |
| **CD8+** | Rho  P | 0.67 0.0002 |  | 0.21 0.31 | 0.03 0.89 | -0.25 0.23 | -0.06 0.77 |
| **CD19+** | Rho  P | 0.30 0.15 | 0.21 0.31 |  | 0.57 0.003 | 0.35 0.09 | 0.67 0.0003 |
| **PDL-1 positive** cell subsets | |  | | | | | |
| **CD4+** | Rho  P | 0.04 0.87 | 0.03 0.89 | 0.57 0.003 |  | 0.76 <0.0001 | 0.71 0.0001 |
| **CD8+** | Rho  P | -0.36 0.08 | -0.25 0.23 | 0.35 0.09 | 0.76 <0.0001 |  | 0.66 0.0003 |
| **CD19+** | Rho  P | -0.03 0.90 | -0.06 0.77 | 0.67 0.0003 | 0.71 0.0001 | 0.66 0.0003 |  |

**Supplementary Table S8 :** Mutual correlations of the frequencies (%) of PD-1 and PD-L1 positive lymphocytes in ALD group. (n=56; Rho- Spearman rank correlation coefficient).

|  | | **PD-1 positive cell subsets** | | | **PDL-1 positive cell subsets** | | |
| --- | --- | --- | --- | --- | --- | --- | --- |
|  |  | **CD4+** | **CD8+** | **CD19+** | **CD4+** | **CD8+** | **CD19+** |
| **PD-1 positive cell subsets** | | | | | | | |
| **CD4+** | Rho  P |  | 0.75 <0.0001 | 0.37 0.005 | 0.06 0.63 | 0.07 0.59 | 0.09 0.53 |
| **CD8+** | Rho  P | 0.75 <0.0001 |  | 0.41  0.002 | 0.01 0.94 | -0.001 0.99 | -0.02 0.88 |
| **CD19+** | Rho  P | 0.37 0.005 | 0.41 0.002 |  | 0.33 0.01 | 0.35 0.009 | 0.39 0.003 |
| **PDL-1 positive cell subsets** | | | | | | | |
| **CD4+** | Rho  P | 0.06 0.63 | 0.01 0.94 | 0.33 0.01 |  | 0.86 <0.0001 | 0.75 <0.0001 |
| **CD8+** | Rho  P | 0.07 0.59 | -0.001 0.99 | 0.35 0.009 | 0.86 <0.0001 |  | 0.77 <0.0001 |
| **CD19+** | Rho  P | 0.09 0.53 | -0.02 0.88 | 0.39 0.004 | 0.75 <0.0001 | 0.77 <0.0001 |  |

**Supplementary Table S8.** An area under the ROC-Curve (AUC) of CD8+PD-L1+cells in predicting mDF above 32.

| **Variable** | **PD-L1 positive cell subsets** | **p value** | **AUC (95% CI)** | **SE^a^** |
| --- | --- | --- | --- | --- |
| **mDF >32** | CD8+ | <0.0001 | 0.88 (0.64- 0.98) | 0.08 |

**Supplementary Figure S1.** Comparison of the frequencies (%) of PD-1 positive CD4+ cells in ALD females with different CTP classes (* p<0.05).

**Supplementary Figure S2.** Comparison of the frequencies (%) of PD-L1 positive CD8+ cells in ALD females with different CTP classes (* p<0.05).

**Supplementary Figure S3.** The ROC of CD8+PD-L1+cells for predicting mDF above 32 in ALD females.

*Youden index J 0.6923; the optimal cut-off threshold >1.08%; Sensitivity 100.00 (95%CI 47.8 – 100.0); Specificity 69.23 (95%CI 38.6 – 90.9); AUC 0.877 (95%CI 0.64- 0.98)

**Supplementary Figure S4.** Flow cytometric sample analysis of PD-1/PD-L1 positive CD4+ (a) and CD8+ (b) cells in a patient with ALD.


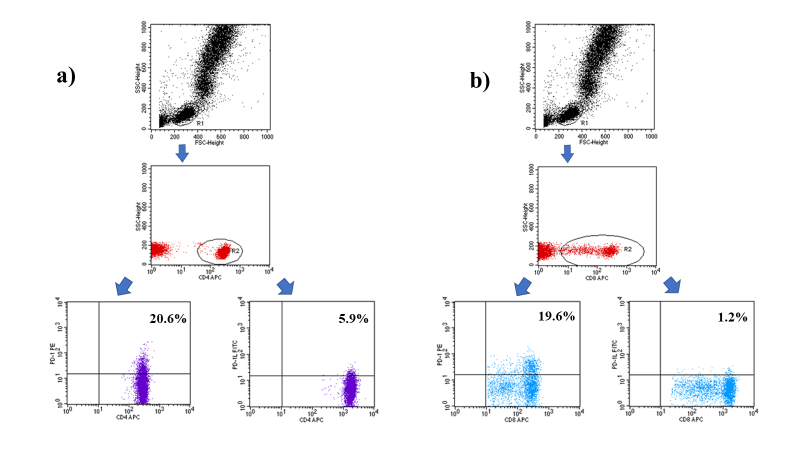


**Supplementary Figure S5.** Flow cytometric sample analysis of PD-1/PD-L1 positive CD19+ cells in a patient with ALD.


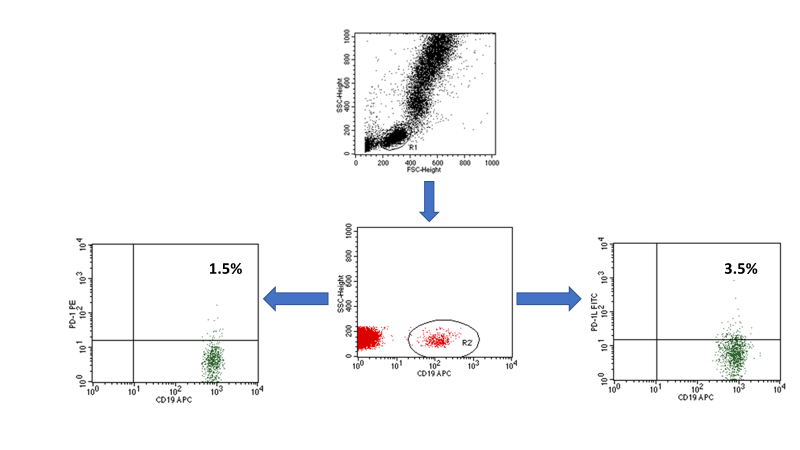

Supplement: File S1 [file peerj-09-10518-s001.docx]
